# Systemic Infusion of Autologous Adipose Tissue-Derived Mesenchymal Stem Cells in Peritoneal Dialysis Patients: Feasibility and Safety

**DOI:** 10.22074/cellj.2019.5591

**Published:** 2018-08-07

**Authors:** Sudabeh Alatab, Soroosh Shekarchian, Iraj Najafi, Reza Moghadasali, Naser Ahmadbeigi, Mohammad Reza Pourmand, Tina Bolurieh, Neda Jaroughi, Gholamreza Pourmand, Nasser Aghdami

**Affiliations:** 1 Urology Research Center, Sina Hospital, Tehran University of Medical Sciences, Tehran, Iran; 2 Department of Regenerative Biomedicine, Cell Science Research Center, Royan Institute for Stem Cell Biology and Technology, ACECR, Tehran, Iran; 3 Urology Research Center, Shariati Hospital, Tehran University of Medical Sciences, Tehran, Iran; 4 Department of Stem Cells and Developmental Biology, Cell Science Research Center, Royan Institute for Stem Cell Biology and Technology, ACECR, Tehran, Iran; 5 Cell-based Therapies Research Center, Digestive Disease Research Institute, Tehran University of Medical Sciences, Tehran, Iran; 6 Department of Pathobiology, School of Public Health, Tehran University of Medical Sciences, Tehran, Iran

**Keywords:** End Stage Renal Disease, Mesenchymal Stem Cell, Peritoneal Dialysis, Peritoneal Fibrosis

## Abstract

**Objective:**

Using mesenchymal stem cells (MSCs) is regarded as a new therapeutic approach for improving fibrotic diseases. the aim of this study to evaluate the feasibility and safety of systemic infusion of autologous adipose tissue-derived MSCs (AD-MSCs) in peritoneal dialysis (PD) patients with expected peritoneal fibrosis.

**Materials and Methods:**

This study was a prospective, open-label, non-randomized, placebo-free, phase I clinical trial. Case group consisted of nine eligible renal failure patients with more than two years of history of being on PD. Autologous AD-MSCs were obtained through lipoaspiration and expanded under good manufacturing practice conditions. Patients received 1.2 ± 0.1×10^6^ cell/kg of AD-MSCs via cubital vein and then were followed for six months at time points of baseline, and then 3 weeks, 6 weeks, 12 weeks, 16 weeks and 24 weeks after infusion. Clinical, biochemical and peritoneal equilibration test (PET) were performed to assess the safety and probable change in peritoneal solute transport parameters.

**Results:**

No serious adverse events and no catheter-related complications were found in the participants. 14 minor reported adverse events were self-limited or subsided after supportive treatment. One patient developed an episode of peritonitis and another patient experienced exit site infection, which did not appear to be related to the procedure. A significant decrease in the rate of solute transport across peritoneal membrane was detected by PET (D/P cr=0.77 vs. 0.73, P=0.02).

**Conclusion:**

This study, for the first time, showed the feasibility and safety of AD-MSCs in PD patients and the potentials for positive changes in solute transport. Further studies with larger samples, longer follow-up, and randomized blind control groups to elucidate the most effective route, frequency and dose of MSCs administration, are necessary (Registration Number: IRCT2015052415841N2).

## Introduction

Peritoneal dialysis (PD) has an increasing rate in last decades, and comprises approximately 11% of dialysis patients, worldwide (1). Accordingly, in Iran we have documented an increase in the number of patients receiving PD over the past 20 years (2). Despite the advantages of this treatment, still the most important challenge is to preserve the peritoneal membrane filtration capacity for a long time (3). Peritoneal fibrosis, as the main cause of filtration capacity loss, has two components: fibrosis and inflammation. Several events such as continuous exposure of the peritoneum to bio-incompatible PD solutions as well as repeated peritonitis episodes provoke activation of various inflammatory, fibrogenic and angiogenic cytokines in peritoneum. The stimulation of these factors leads to progressive detachment of the mesothelial cell layer and its transformation into fibroblastoid cells, submesothelial fibrosis, extensive vasculopathy and ultimately ultrafiltration failure (UFF) and encapsulating peritoneal sclerosis (EPS) (4). 

Mesenchymal stem cells (MSCs) are a heterogeneous adult stem cell population with important immunoregulatory and anti-fibrotic activities (5-7). They have the potential to differentiate into mesodermal and non-mesodermal lineages, secrete factors that favoring tissue remodeling, and might even successfully escape immune recognition (7, 8). Based on their properties, researchers have already performed pioneering clinical studies evaluating the safety and possible efficacy of MSC administration in various diseases including renal failure (9-12). For instance, Makhlough et al. (11) showed the safety and tolerability of one-time intravenous (IV) transplantation of autologous bone marrow-derived MSCs in autosomal dominant polycystic kidney disease patients. 

Regarding PD, many preclinical studies have been performed to evaluate the effects of stem cell therapy in PD experimental models (13-17). The overall findings of these researches have been that use of stem cells is associated with improved peritoneal fibrosis including attenuation of submesothelial thickness and collagen deposition, inflammation, angiogenesis, fibrosis and also improvement of peritoneal permeability function. This improvement was indicated by higher ultrafiltration, lower glucose transport and better solute permeability. No adverse events following MSC administration were reported by these studies (17). In addition, the mechanisms, by which the beneficial effects of MSCs were exerted were different. Some studies showed the contribution of stem cells in the process of peritoneal repair and mesothelial remodeling (13), while others provided evidences supporting the paracrine activities of MSCs, involving the production of growth factors and anti-inflammatory cytokines. These factors produced by MSCs were shown to promote repair, independent of trans-differentiation of MSCs into functional peritoneal mesothelial cells (14-16). However the gap between experimental studies and clinical applications still exists, as none of these experimental studies have been translated into clinical cases yet.

Therefore, to continue the translation of experimental research into clinical research, we have designed a phase I clinical study to evaluate the feasibility and safety of administration of autologous adipose tissue-derived MSCs (AD-MSCs) in renal failure patients. We have previously shown that UFF is a risk factor for developing severe peritoneal fibrosis (18), thus we sought to evaluate the safety of administration of MSCs in patients who suffer from UFF. 

## Materials and Methods

### Materials and Methods

This prospective study was a 6-month, open-label, non- randomized, phase I trial to evaluate the safety of single IV infusion of autologous AD-MSCs in PD patients. The study was conducted in Urology Research Center of Tehran University of Medical Sciences and Cell Science Research Center of Royan Institute, Tehran, Iran. All procedures performed in studies involving human participants were in accordance with the ethical standards of the institutional and research committee and with the 1964 Helsinki Declaration. The Institutional Review Board and local Ethics Committee of Tehran University of Medical Sciences (code: 93-03-47-27290-146850) and Royan Institute (IR.ACECR.ROYAN.REC.1395.2294000123) approved this study. All participants gave informed written consent. The IRCT of this study is (IRCT2015052415841N2) which was conducted from October 2015 to March 2017. An external trial monitor was enlisted to assess the conduct of the trial and accuracy of the data.

### Patient selection

The study population consisted of patients between 18 and 70 years of age, who were on continuous ambulatory peritoneal dialysis (CAPD) and referring to PD centers of Shafa hospital, which is affiliated with Tehran University of Medical sciences. The eligibility criteria were: having been on CAPD for at least two years, having UFF [ultrafiltration (UF)<400 mL after a 4-hour dwell duration with a 4.25% dextrose-based PD fluid], and being available for follow-ups. Patients were excluded if they were pregnant or had plans to become pregnant, were candidates for kidney transplantation, had consumed immunosuppressive drugs, had a confirmed cancer, had coagulation disorders, or had a history of hospitalization or hemodialysis two months prior to study entry. Potential participants underwent preliminary screening by the study doctor and those found to be eligible were invited to attend a baseline assessment. In the case of eligibility, formal written consent form was given to each patient to sign. Patients were also provided with the study doctor’s contact details to discuss any concerns before, during or after the procedures.

### Isolation and expansion of adipose-derived mesenchymal stem cells

Human adipose tissue (150-250 mL) was collected under local anesthesia followed by lipo-aspiration surgery performed by a plastic surgeon at Royan Institute. All patients were discharged 2 hours after the procedure, but had phone follow up for 2 days. The lipo-aspirated samples were collected in sterile tubes containing phosphate- buffered saline (PBS)+1% penicillin/streptomycin and were transferred to our laboratory on ice. The samples were washed 3-4 times with sterile water. They were then enzymatically digested with 0.075% collagenase type I at 37°C for 2 hours in an incubator with intermittent spinning. The procedure was followed by adding alpha modified eagle medium (a-MEM, Gibco, Germany), in a volume twice the volume of enzyme, to the tube and centrifugation at 1500 rpm for 5 minutes. Next, the pellet was diluted in 1-2 ml of a-MEM, passed through a 40-µm mesh filter and plated at a density of 1×10^6^/cm^2^ in a 25-T culture flask in a-MEM supplemented with 1% penicillin/ streptomycin (Gibco, Germany), 10% Hyclone defined fetal bovine serum (FBS, Thermo Scientific, USA), and 1% L-glutamine (Gibco, Germany) at 37°C . The medium was changed every 4 days till 90% confluency was achieved. The cells were passaged up to three times. Finally, cells were washed with PBS, detached from the flask using Trypsin/EDTA (0.2%), suspended in physiological serum (Gibco, Germany) and loaded into 10-ml sterile syringes.

We performed the colony-forming unit (CFU) assay to determine the proliferation potentials of the cultured AD-MSCs. The immunophenotype of AD-MSCs were characterized for each patient using 2×10^5^ MSCs from passage 2 cells. Next, the cells were incubatedin thedark for 20 minutes at room temperature, with phycoerythrin (PE)conjugated CD105, CD44, CD73, 11b, CD34 (Becton Dickinson, Franklin Lakes, NJ, USA) and fluorescein isothiocyanate (FITC)-conjugated CD 90, CD45 (Dako, Glostrup, Denmark), then washed three times with PBS. Staining with nonspecific mouse IgG1-FITC/IgG1PE and IgG2a-FITC was used as negative control. The fluorescent labeled cells were analyzed on a FACSscan flow cytometer (BD FACS Caliber, BD Biosciences, San Jose CA, USA) using MDI 2.9 software (Table 1). 

**Table1:** Parameters of infused mesenchymal stem cells in PD patients


Cell parameters	Pt ID:01	Pt ID:02	Pt ID:03	Pt ID:04	Pt ID:05	Pt ID:06	Pt ID:07	Pt ID:08	Pt ID:09

Count (n)	52×10^6^	80×10^6^	80×10^6^	80×10^6^	80×10^6^	80×10^6^	80×10^6^	95×0^6^	85×10^6^
Viability (%)	98	93	97	100	95	97	95	97	97
Markers (%)									
CD90	99.4	99.8	99.1	99	99.9	99.5	95.8	93.1	97.2
CD105	99.5	95.7	99.2	99.6	99.2	99.4	85.5	76.3	94
CD73	98.6	98.1	98.5	95.1	96.9	97.7	65.4	81.1	76.8
CD44	95.7	95.2	92.2	97.2	92.6	97.5	92.2	88.7	85.2
CD11b	27.6	30.4	28.8	2.1	16.8	11.1	3.5	2.4	15.2
CD34	2.07	2.37	1.41	0.34	3.26	4.02	0.4	0.52	1.9
CD45	0.01	0.03	0.4	0.02	0.01	0.06	0.27	1.6	1.06
Microbial test BM	Negative	Negative	Negative	Negative	Negative	Negative	Negative	Negative	Negative
Microbial test MNC	Negative	Negative	Negative	Negative	Negative	Negative	Negative	Negative	Negative
Microbial test UM	Negative	Negative	Negative	Negative	Negative	Negative	Negative	Negative	Negative
Microbial test bulk	Negative	Negative	Negative	Negative	Negative	Negative	Negative	Negative	Negative
Microbial test final	Negative	Negative	Negative	Negative	Negative	Negative	Negative	Negative	Negative
Cytogenetic report									
Mycoplasma test	Negative	Negative	Negative	Negative	Negative	Negative	Negative	Negative	Negative
LAL test (EU/ml)	< 0.125	< 0.125	< 0.125	< 0.125	< 0.125	< 0.125	< 0.125	< 0.125	< 0.125
Patient viral test									
Anti-HCV Ab	Negative	Negative	Negative	Negative	Negative	Negative	Negative	Negative	Negative
Anti-HIV 1,2	Negative	Negative	Negative	Negative	Negative	Negative	Negative	Negative	Negative
HBS Ag	Negative	Negative	Negative	Negative	Negative	Negative	Negative	Negative	Negative
Anti-HBC Ab	Negative	Negative	Negative	Negative	Negative	Negative	Negative	Negative	Negative
Anti-HBS Ab	Reactive	Negative	Negative	Negative	Negative	Negative	Negative	Negative	Negative
Anti-HTLV	Negative	Negative	Negative	Negative	Negative	Negative	Negative	Negative	Negative


Cell count by neobar slide and nucleocounter; Viability test by nucleocounter and trypan blue; Cell markers by flow cytometry and analyzed by FlowJosoftware; Microbial tests by the BACTEC system, Mycoplasma by nested PCR; LAL by the endotoxin test; Viral tests were performed using ELISA. PD; Peritoneal dialysis, LAL; limulus Amebocyte Lysate, HCV; hepatitis C virus , HIV; Human immunodeficiency virus, HBS; Hepatitis B surface antigen, HBC; Hepatitis B core antibody , HTLV; Human T-lymphotropic virus , BM; Bone marrow, MNC; Mono nuclear cell, and UM; Upper medium.

### Quality control tests

The quality control tests included the analysis of microbial tests, limulus amebocyte lysate (LAL) gel clot assays, mycoplasma detection and karyotyping. These procedures were performed according to recommendationsfor cell and tissue therapy promotion and validation tests of the Iranian Health Ministry Pharmacopoeia Commission and the Department of Health and Human Services Food and Drug Administration.

### Adipose tissue-derived mesenchymal stem cells administration

The fresh suspension of the cells in 50 ml normal saline was infused through cubital vein under sterile conditions according to our infusion protocol. After infusion, patients were monitored closely in a hospital setting for 4 hours and their vital signs as well as immediate adverse events in the site of infusion were monitored.

### Endpoint measures 

The primary endpoint was feasibility, safety and tolerability of MSC infusion. A detailed explanation of assessment schedule is presented in Table 2.

**Table 2:** Assessment schedule of the study


Assessment	Baseline (v1)	Week 3 (v2)	Week 6 (v3)	Week 12 (v4)	Week 16 (v5)	Week 24 (v6)

Time window	-30 to -7 day	± 4 day	± 4 day	± 4 day	± 4 day	± 4 day
Informed consent	X					
Vital signs	X	X	X	X	X	X
Medical history	X					
Physical exam	X	X	X	X	X	X
Lipoaspiration	X					
CBC	X	X	X	X	X	X
Electrolyte, Ca, P, FBS levels	X	X	X	X	X	X
Liver function test	X	X	X	X	X	X
Renal function test	X	X	X	X	X	X
Lipid profile	X	X	X	X	X	X
coagulation parameters	X	X	X	X	X	X
iron, ferrittin, TIBC levels	X	X	X	X	X	X
PTH levels	X	X	X	X	X	X
Alkp levels	X	X	X	X	X	X
Albumin levels	X	X	X	X	X	X
ESR	X	X	X	X	X	X
24 hour urine volume	X	X	X	X	X	X
24 hour UF volume	X	X	X	X	X	X
Cr clearance	X			X		X
D/P cr	X			X		X
D/P urea	X			X		X
D/D0 glucose	X			X		X
Kt/v	X			X		X
nPCR	X			X		X
Peritoneal equilibration test	X			X		X
AE assessment		X	X	X	X	X
SAE assessment		X	X	X	X	X


v1; Baseline visit before infusion, v; Visit, CBC; Complete blood count, FBS; Fasting blood sugar, Ca; Calcium, P; Phosphorous, PTH; Parathyroid hormone, ESR; Erythrocyte sedimentation rate, AlK-p; Alkaline phosphatase, TIBC; Total iron binding capacity, UF; Ultrafiltration, cr; Creatinin, D/P cr; Dialysate toplasma ratio of creatinin, D/P urea; Dialysate to plasma ratio of urea, D/D0 glucose; Dialysate glucose at 4 hours’ dwell time to dialysis glucose at time 0, nPCR; Normalized protein catabolic rate, HIV; Human immunodeficiency virus, HCV; Hepatitis C virus, HBV; Hepatitis B virus, HTLV; Human T-lymphotropicvirus, AE; Adverse event, and SAE; Serious adverse event.

### MSCs in PD Patients

The primary endpoint was evaluated on the basis of the occurrences of serious adverse events (SAEs), AEs, and laboratory abnormalities according to the Common Terminology Criteria for Adverse Events (CTCAE) version 4.0. They were obtained through physical examination, biochemical assays and peritoneal solute transport tests (Table 2). The time points of visits were selected based on previous studies evaluating the effects and also the safety of MSCs in different diseases (12). Peritoneal solute transport parameters were measured through performing a peritoneal equilibration test (PET) at V1, V4 and V6 according to previous protocols (19). Dialysis adequacy parameters, including Kt/V, creatinine clearance and normalized protein catabolic rate (nPCR) were calculated based on the analysis of a 24-hour collection of dialysis solution and urine with the PD Adequest 2.0 for Windows program (Baxter Healthcare Co, Deerfield, Illinois, USA). 

Patients were required to report any adverse events related to the treatment they had received in the trial and also changes indicative of peritonitis, such as cloudy peritoneal effluent and abdominal pain during the follow-up visits.

Mean of 24-hour UF and urine volume records within one week before baseline or follow-up visits were defined as UF or urine volume of that visit. To avoid the effects of daily dialysis volume on UF, the total daily volume of PD fluid was not changed during the follow-up period. 

### Statistical analysis 

The continuous variables are presented as mean ± SD. The categorical variables are expressed as absolute values and frequencies. General Linear Model was used to calculate repeated measures of ANOVAs for functional and safety parameter from baseline at the follow up visits for each group. P<0.05 were considered statistically significant. Analyses were performed using SPSS software (version 16.0, NY, USA). 

## Results

For this study we originally recruited 10 eligible patients as our case group, however patient number 10 refused to participate after liposuction and therefore was excluded from the study. The other 9 patients finished the trial until the last follow up. The summarized characteristics of the recruited subjects are presented in Table 3. Each participant received a single IV infusion of autologous AD- MSCs (mean 1.2×10^6^, range 0.9-1.4). The clinical characteristics of each subject is presented in Table 4. 

### Safety of mesenchymal stem cell infusion

No SAEs occurred throughout the trial. No acute infusion-related toxicity was observed during or immediately after MSC infusion. A total of 14 minor AEs were reported by 6 patients that were potentially related to the procedure. These AE’s included five abdominal bruises induced by lipo-aspiration, which lasted for 5-10 days and resolved spontaneously, four mild to moderate cases of abdominal pains that developed following lipo-aspiration, which lasted for less than 48 hours and resolved spontaneously, one grade 2 phlebitis at the site of venous access (right arm), which resolved by using analgesic, application of hot compress and elevation of extremity within 5 days, and finally three flank pains and one headache, all of which resolved by using analgesics within 2 days after infusion. Abdominal lipo-aspiration sample points healed in less than7 days post-surgery for all patients. Detailed explanation of the AEs is presented in Table 5.

### Peritonitis 

One patient developed one episode of peritonitis during the follow up period (case ID#03, in the 5th month of follow-up). This patient presented with abdominal pain and cloudy effluent. Culture of dialysis fluid was positive for E-coli and the patient responded to amikacin (100 mg/IP) and ceftazidim (1 g/IP). In case ID#4, in the 1st month of follow-up, we noted exudates from the exit site, but the patient had good inflow and outflow and clear effluent. This patient was diagnosed with exit site infection with or without tunnel infection. The result of ultrasound of the tunnel was negative and the patient responded to amikacin100 mg/day, IP, ceftazidime 1 g/day, IP, Rifampin 300 mg/BID.

### Monitoring biochemical and peritoneal membrane parameters 

None of the hematological and biochemical parameters significantly changed over time in the participants. The 24-hour UF collection showed a significant increase (20.5%, P=0.01), while 24hour urine sample volume showed a non-significant decrease (41%). A significant decrease in body mass index (BMI) was also observed (Table 6). Moreover, we observed a decline in the rate of solute transport across peritoneal membrane (D/Pcr, P=0.02, 0.76 vs. 0.72), however the other parameters regarding the dialysis adequacy and solute transport did not change during the follow-up.

**Table 3:** Characteristics of enrolled subjects at the time of enrollment into study


Parameter		Case group

Age (Y) (Mean ± SD, minimum-maximum)		55.6 ± 11.9, 43-70
Sex (male: female)		3:6
Cause of ESRD		
	Diabetic nephropathy	2
	Hypertensive nephropathy	3
	Poly cystic kidney disease	1
	Recurrent UTI	1
	Glumeronephritis	0
	Unknown	2
Duration of ESRD (month) (Mean ± SD, minimum-maximum)		125.4 ± 76, 36-278
Previous HD (yes: no)		3:6
Duration of previous HD (month) (Mean ± SD, minimum-maximum)		36.7 ± 66, 0-193
Previous Tx (yes: no)		3:6
Duration of PD (month) (Mean ± SD, minimum-maximum)		77.1 ± 41.4, 24-124
Comorbidity		
	Diabetes (yes: no)	4:5
	Hypertension (yes: no)	7:2
Transport status		
	Low	0
	Low average	0
	High average	6
	High	3
Anuria (yes: no)		6:3
Ultrafiltration (ml/day) (Mean ± SD, minimum-maximum)		1216.6 ± 573.4, 300-2000
Weight (kg) (Mean ± SD)		67.2 ± 12.1
BMI (kg/m^2^) (Mean ± SD)		26.9 ± 5.3
SBP (mmHg) (Mean ± SD, minimum-maximum)		128.8 ± 18.3, 110-170
DBP (mmHg) (Mean ± SD, minimum-maximum)		80.5 ± 13.3, 65-110
MSCs administration (cell/kg)		1, 191, 631 ± 132, 327, 941, 176-1, 363, 636


ESRD; End-stage renal disease, UTI; Urinary tract infection, HD; Hemodialysis, Tx; Kidney transplant, PD; Peritoneal dialysis, BMI; Body mass index, SBP; Systolic blood pressure, DBP; Diastolic blood pressure, and MSCs; Mesenchymal stem cells.

**Table 4:** Clinical characteristics of the case group at the time of enrollment


Case ID	01	02	03	04	05	06	07	08	09
Parameter									

Age (Y)	47	70	69	43	69	63	44	44	52
Sex (M/F)	F	F	M	F	M	F	F	F	M
ESRD duration (month)	217	96	36	278	121	121	124	68	66
PD duration (month)	81	96	36	25	121	121	124	24	66
DM	No	No	Yes	No	No	Yes	Yes	Yes	No
HTN	No	Yes	Yes	No	Yes	Yes	Yes	Yes	Yes
Cause of ESRD	Unknown	PKD	HTN	Rec.UTI	HTN	DM	Unknown	DM	HTN
Weight (kg)	43	63	73	67	66	63	85	81	64
BMI (kg/m^2^)	19.1	24.6	27.8	26.2	21.6	27.3	36.3	33.7	25.6
SBP (mmHg)	120	110	130	110	120	140	130	130	170
DBP (mmHg)	65	70	80	70	80	80	90	80	110
Anuria (yes/no)	Yes	Yes	No	Yes	No	No	Yes	Yes	Yes
Overnight fluid	Ico	Ico	4.25% Dextrose	Ico	Ico	4.25% Dextrose	Ico	Ico	Ico
Transport type	H	H	HA	HA	HA	H	HA	HA	HA
24 hours UF (ml)	1300	1600	800	850	1200	300	2000	2000	900
GFR (ml/minutes/1.73 m^2^)	0	0	3.55	0	2.94	1.58	0	0	0
D/P cr	0.93	0.85	0.76	0.69	0.74	0.88	0.74	0.69	0.7
N of exchange/24 hours	5	4	4	5	4	4	4	5	4
No of cell/kg	1, 155, 555.6	1, 269, 841.3	1, 066, 666.7	1, 290, 322.6	1, 363, 636.4	1, 269, 841.3	941, 176.5	1, 117, 647.1	1, 250, 000.0
Medication	Eprex (4000 unit/3 week), calcium carbonate, nephrovite	Erythropoietin, nephrovite, ASA (80 mg), atorvastatin (40 mg/day), losartan (50 mg/BID)	Eprex (4000 unit/8 week), calcium carbonate, folic acid, ASA (80 mg), captopril (50 mg/day), losartan (25 mg/BID), amlodipine (5 mg/day), furosemide (60 mg/TID), atorvastatin (20 mg/day), allopurinol (100 mg/day), insulin	Erythropoietin, calcium carbonate, Rocaltrol,	Erythropoietin, calcium carbonate, folic acid, B complex, renagel (8 mg/day), furosemide (40 mg/BID), Allopurinol (100 mg/day)	Erythropoietin, nephrovite, calcium carbonate, rocaltrol, vitamin c, carnitine, spironolactone (50 mg/day), furosemide (80 mg/BID)	Erythropoietin, calcium carbonate, rocaltrol, folic acid, vitamin c, losartan (50 mg/BID), atorvastatin (20 mg/BID), insulin	Erythropoietin, calcium carbonate, rocaltrol, vitamin D, ASA (80 mg), allopurinol (100 mg/day), losartan (50 mg/ BID), metoral (100 mg/day), spironolactone (50 mg/day), insulin	Erythropoietin, calcium carbonate, vitamin D, ASA (80 mg), folic acid, Amlodipin (5 mg/BID), valsacor (160 mg/BID), spironolactone (25 mg/day)


ESRD; End-stage renal disease, Rec, UTI; Recurrent urinary tract infection, PD; Peritoneal dialysis, DM; Diabetes mellitus, HTN; Hypertension, BMI; BodyMass Index, SBP; Systolic blood pressure, DBP; Diastolic blood pressure, UF; Ultrafiltration, Ico; Icodextrin, PKD; Polycystic kidney disease, F; Female, M; Male, GFR; Glomerular filtration rate, D/P cr; Dialysate to plasma ratio of creatinin, H; High, and HA; High average.

**Table 5:** Safety assessment of mesenchymal stem cells infusion in study subjects


Serious adverse events (SAE)	Pt 1	Pt 2	Pt 3	Pt 4	Pt 5	Pt 6	Pt 7	Pt 8	Pt 9

Death	n	n	n	n	n	n	n	n	n
Required hospitalization	n	n	n	n	n	n	n	n	n
Life-threatening	n	n	n	n	n	n	n	n	n
Any	n	n	n	n	n	n	n	n	n
Total	0	0	0	0	0	0	0	0	0
Adverse events									
Blood and lymphatic system disorders (y or n, grade)									
Leukocytosis	n	n	n	n	n	n	n	n	n
Lymph node pain	n	n	n	n	n	n	n	n	n
Any	n	n	n	n	n	n	n	n	n
Cardiac disorders (y or n, grade)					
Acute coronary syndrome	n	n	n	n	n	n	n	n	n
Any	n	n	n	n	n	n	n	n	n
Ear and labyrinth disorders (y or n, grade)					
Ear pain	n	n	n	n	n	n	n	n	n
Vertigo	n	n	n	n	n	n	n	n	n
Any	n	n	n	n	n	n	n	n	n
Endocrine disorders (y or n, grade)					
Hypothyroidism	n	n	n	n	n	n	n	n	n
Hyperparathyroidism	n	n	n	n	n	n	n	n	n
Any	n	n	n	n	n	n	n	n	n
Eye disorders (y or n, grade)					
Conjunctivitis	n	n	n	n	n	n	n	n	n
Dry eye	n	n	n	n	n	n	n	n	n
Any	n	n	n	n	n	n	n	n	n
Gastrointestinal disorders (y or n, grade)					
Abdominal pain	n	y, 1	n	y, 1	y, 1	n	n	y, 1	n
Dyspepsia	n	n	n	n	n	n	n	n	n
Diarrhea	n	n	n	n	n	n	n	n	n
Nausea	n	n	n	n	n	n	n	n	n
Any	n	n	n	n	n	n	n	n	n
General disorders and administration site conditions (y or n, grade)									
Fatigue	n	n	n	n	n	n	n	n	n
Fever	n	n	n	n	n	n	n	n	n
**Table 5: Continued**									
**Serious adverse events (SAE)**	Pt 1	Pt 2	Pt 3	Pt 4	Pt 5	Pt 6	Pt 7	Pt 8	Pt 9
Pain	n	n	n	n	n	n	n	n	n
Infusion site reaction	n	n	n	n	n	n	n	n	y, 2
Any	n	n	n	n	n	n	n	n	n
Hepatobiliary disorders (y or n, grade)					
Hepatic failure	n	n	n	n	n	n	n	n	n
Any	n	n	n	n	n	n	n	n	n
Immune system disorders (y or n, grade)					
Allergic reaction	n	n	n	n	n	n	n	n	n
Anaphylaxis	n	n	n	n	n	n	n	n	n
Any	n	n	n	n	n	n	n	n	n
Infections and infestations (y or n, grade)					
Bladder infection	n	n	n	n	n	n	n	n	n
Catheter related infection	n	n	n	y, 2	n	n	n	n	n
Peritoneal infection	n	n	y, 3	n	n	n	n	n	n
Urinary tract infection	n	n	n	n	n	n	n	n	n
Any	n	n	n	n	n	n	n	n	n
Procedural complications (y or n, grade)					
Intraoperative skin injury	n	y, 2	n	y, 2	y, 2	n	n	y, 2	y, 2
Any	n	n	n	n	n	n	n	n	n
Increased alanine aminotransferase	n	n	n	n	n	n	n	n	n
Increased alkaline phosphatase	n	n	n	n	n	n	n	n	n
Increased aspartate aminotransferase	n	n	n	n	n	n	n	n	n
Creatinine increased	n	n	n	n	n	n	n	n	n
Hemoglobin increased	n	n	n	n	n	n	n	n	n
Lymphocyte count decreased	n	n	n	n	n	n	n	n	n
Lymphocyte count increased	n	n	n	n	n	n	n	n	n
Platelet count decreased	n	n	n	n	n	n	n	n	n
Weight gain	n	n	n	n	n	n	n	n	n
Weight loss	n	y, 1	y, 1	n	n	n	n	n	n
Any	n	n	n	n	n	n	n	n	n
Metabolism and nutrition disorders (y or n, grade)					
Anorexia	n	n	n	n	n	n	n	n	n
Hyperkalemia	n	n	n	n	n	n	n	n	n
Hyponatremia	n	n	n	n	n	n	n	n	n
Hypokalemia	n	n	n	n	n	n	n	n	n
Hypoglycemia	n	n	n	n	n	n	n	n	n
Hypoalbuminemia	n	n	n	n	n	n	n	n	n
New-onset diabetes	n	n	n	n	n	n	n	n	n
Any	n	n	n	n	n	n	n	n	n
**Table 5: Continued**									
**Serious adverse events (SAE)**	Pt 1	Pt 2	Pt 3	Pt 4	Pt 5	Pt 6	Pt 7	Pt 8	Pt 9
Musculoskeletal and connective tissue disorders (y or n, grade)					
Arthritis	n	n	n	n	n	n	n	n	n
Arthralgia	n	n	n	n	n	n	n	n	n
Back pain	n	n	n	n	n	n	n	n	n
Flank pain	n	n	n	y,1	y,1	n	n	n	y,1
Any	n	n	n	n	n	n	n	n	n
Neoplasms benign, malignant and unspecified include cysts and polyps (y or n, grade)					
Benign	n	n	n	n	n	n	n	n	n
Malignant	n	n	n	n	n	n	n	n	n
Any	n	n	n	n	n	n	n	n	n
Nervous system disorders (y or n, grade)					
Cognitive disturbance	n	n	n	n	n	n	n	n	n
Dizziness	n	n	n	n	n	n	n	n	n
Headache	n	n	n	y,1	n	n	n	n	n
Seizure	n	n	n	n	n	n	n	n	n
Any	n	n	n	n	n	n	n	n	n
Psychiatric disorders (y or n, grade)					
Anxiety	n	n	n	n	n	n	n	n	n
Confusion	n	n	n	n	n	n	n	n	n
Depression	n	n	n	n	n	n	n	n	n
Any	n	n	n	n	n	n	n	n	n
Renal and urinary disorders (y or n, grade)					
Urinary frequency	n	n	n	n	n	n	n	n	n
Any	n	n	n	n	n	n	n	n	n
Reproductive system and breast disorders (y or n, grade)					
Breast pain	n	n	n	n	n	n	n	n	n
Any	n	n	n	n	n	n	n	n	n
Respiratory, thoracic, and mediastinal disorders (y or n, grade)					
Allergic rhinitis	n	n	n	n	n	n	n	n	n
Cough	n	n	n	n	y,1	n	n	n	n
Dyspnea	n	n	n	n	n	n	n	n	n
Any	n	n	n	n	n	n	n	n	n
Skin and subcutaneous tissue disorders (y or n, grade)					
Pruritus	n	n	n	n	n	n	n	n	n
Eczema	n	n	n	n	n	n	n	n	n
Any	n	n	n	n	n	n	n	n	n
Vascular disorders (y or n, grade)					
Any	n	n	n	n	n	n	n	n	n
Total	0	3	2	5	4	0	0	2	3


Pt; Patient, y; Yes, n; No. Adverse events were categorized according to the Common Terminology Criteria for Adverse Events (CTCAE) Version 4.0.

**Table 6:** Clinical and laboratory parameters at baseline and follow-ups


Parameter	V1	V2	V3	V4	V5	V6	P

Weight (kg)	67.2 (12)	67.8 (11)	68.1 (11)	67.2 (11)	67.4 (11)	65.4 (11)	0.006*
BMI (kg/m^2^)	26.9 (5.3)	27.1 (5.1)	25 (3.7)	24.6 (3.7)	24.6 (3.7)	23.8 (3.6)	0.001*
Systolic BP (mmHg)	128.8 (18.3)	130 (18)	134.4 (26.5)	135 (27.6)	124.4 (18)	125 (26.9)	0.5
Diastolic BP (mmHg)	80.5 (13)	77.2 (13)	80 (18)	83.3 (13)	75.5 (13)	73.3 (14)	0.4
WBC (/µl)	7611 (1693)	7485 (1693)	7214 (1197)	6144 (1639)	6408 (2556)	7144 (2060)	0.2
Hb (g/dl)	10.9 (1.3)	11.2 (1.5)	10 (3.8)	10.6 (1.7)	10.6 (1.6)	11.2 (2.3)	0.3
Plate (/µl)	201, 888 (57,444)	205,375 (62,848)	191, 857 (44,069)	200, 666 (51, 320)	238, 142 (61, 224)	225, 777 (63, 357)	0.1
ESR (mm/h)	72.5 (18)	55.8 (20)	58.1 (22)	69.1 (23)	68.2 (7)	60.3 (15)	0.1
Iron (g/dl)	64.3 (26)	61.5 (25)	52.7 (21)	59.7 (23)	60 (17)	56.5 (26)	0.8
Ferr (µg/l)	641 (415)	677 (403)	579 (384)	679 (299)	548 (370)	778 (558)	0.3
TIBC (micg/dl)	298.1 (90)	238.2 (45)	231.5 (41)	241.8 (49)	232.7 (41)	250.6 (29)	0.1
FBS (mg/dl)	128.3 (54)	107.5 (25)	97.8 (20)	102.2 (9)	99.1 (37)	115.7 (42)	0.1
BUN (mg/dl)	108.8 (38)	98.4 (35)	106.1 (22)	113.8 (25)	102.3 (28)	93.2 (25)	0.2
Cr (mg/dl)	10.5 (3.5)	11 (3.6)	10.8 (3.6)	11.1 (3.2)	10.7 (3.3)	10.8 (3.2)	0.7
UA (mg/dl)	6 (1)	6 (1.2)	5.9 (1.8)	5.5 (1.2)	5.7 (1.4)	5.6 (1)	0.2
ALT (U/l)	17.4 (6.5)	16.6 (5.8)	16.1 (67)	14.2 (5)	16.2 (6.4)	20.7 (16)	0.4
AST (U/l)	16.3 (4.6)	16.7 (6.6)	16 (7.1)	18.8 (9)	15.5 (6.5)	17.7 (7.9)	0.6
Na (mEq/l)	133.5 (3)	133.7 (4)	135 (4)	134.3 (4)	134 (1.9)	135.8 (2)	0.3
K (mEq/l)	4 (0.6)	4.1 (0.5)	4.2 (0.6)	4.2 (0.6)	4.1 (0.5)	4.3 (0.4)	0.3
Ca (mg/dl)	8.6 (2.8)	9.2 (1.3)	9.1 (1.2)	9.5 (1)	9.4 (1)	9.7 (0.8)	0.5
Ph (mg/dl)	4.8 (1.4)	4.4 (0.7)	4.6 (0.9)	4.8 (1.2)	4.8 (1.3)	4.7 (0.9)	0.3
Alkp (IU/l)	587 (830)	591 (809)	639 (869)	557 (756)	475 (541)	366 (269)	0.2
PTH (pg/ml)	422 (535)	550 (954)	510 (738)	408 (652)	417 (539)	306 (418)	0.3
Cholesterol	168 (31)	169 (44)	180 (57)	159 (31)	150 (27)	158 (21)	0.3
Triglyceride (mg/dl)	183 (185)	199 (252)	133 (65)	162 (116)	100 (36)	153 (87)	0.4
HDL (mg/dl)	49.6 (16)	55 (17)	46.8 (15)	48.3 (15)	54.7 (6)	50 (12)	0.4
LDL (mg/dl)	81.2 (28)	63.2 (10)	84.5 (44)	79.1 (24)	71 (18)	76.3 (16)	0.2
Alb (g/dl)	3.7 (0.3)	3.8 (0.3)	3.7 (0.3)	3.8 (0.3)	3.5 (0.3)	3.8 (0.3)	0.2
UF24 hours (ml)	1216 (573)	1455 (545)	1577 (526)	1616 (560)	1461 (572)	1466 (532)	0.01^*^
UV24 hours (ml)	266 (409)	288 (448)	322 (556)	255 (403)	244 (418)	155 (278)	0.4
D/P Cr	0.77 (0.09)	NA	NA	0.73 (0.08)	NA	0.73 (0.08)	0.02^*^
D/P urea	0.86 (0.04)	NA	NA	0.86 (0.04)	NA	0.84 (0.03)	0.4
Dt/D0 glucose	0.26 (0.07)	NA	NA	0.26 (0.04)	NA	0.29 (0.04)	0.9
Total Kt/V	1.8 (0.3)	NA	NA	1.7 (0.2)	NA	1.8 (0.3)	0.5
Total CrCl	55.3 (10)	NA	NA	53.2 (9)	NA	53.9 (10)	0.7
nPCR	0.71 (0.2)	NA	NA	0.70 (0.1)	NA	0.63 (0.2)	0.2


Data are presented as mean (SD), P<0.05 were significant. BMI; Body mass index, BP; Blood pressure, FBS; Fasting blood sugar, PTH; Parathyroid hormone, WBC; White blood cells, Hb; Hemoglobin, ESR; Erythrocyte sedimentation rate, TIBC; Total iron binding capacity, BUN; Blood urea nitrogen, Cr; Creatinin, UA; Urinalysis, ALT; Alanine aminotransferase, AST; Aspartate aminotransferase, Na; Sodium, K; Potassium, Ph; Phosphorus, Alkp; Alkaline phosphatase, HDL; High-density lipoproteins, LDL; Low-density lipoproteins, Alb; Albumi, UF; Ultrafiltration, UV; Urine volume, D/P cr; Dialysate to plasma ratio of creatinin, Dt/D0 glucose; Dialysate glucose at 4 hours’ dwell time to dialysis glucose at time 0, and nPCR; Normalized protein catabolic rate.

## Discussion

The present study, which was designed as a phase I clinical trial showed that IV injection of autologous AD-MSCs in CAPD patients with UFF is safe and well- tolerated. To our knowledge, this is the first clinical trial that provides evidence for safety of systemic infusion of autologous MSCs in PD patients. The most important AEs was development of grade 2 phlebitis in one patient, which might be related to the acute inflammatory reactions of some patients to particular preparations of stem cells (12). Assessing the hematological and systemic biochemical parameters showed the stability of these parameters over time. The only significant change in the treated group was attenuation of BMI, which is probably attributed to a decrease in the degree of edema due to a significant increase in ultrafiltration rate. A significant weight loss in patients over time could also support this observation. The attenuation of the urine volume seen in this study could also be attributed to an increased ultrafiltration volume, as it is expected that patients who have more ultrafiltration might have less urine volume. Although we should note that only 3 patients had urine output and therefore we cannot generalize this assumption. 

We used adipose tissue-derived MSCs for several reasons. Our previous experiments have shown that bone marrow-derived MSCs from ESRD patients can be expanded up to a limited number (data not published), which might be due to dysplasia of bone marrow caused by azotemia in these patients. Nonetheless, a large number of MSCs can be easily isolated from fat tissue in these patients. Moreover, while exposure of patients with renal failure to uremic toxins over long periods of time may affect the viability and regenerative capacity of MSCs compared to those in patients with normal renal function (20, 21), Roemeling-van Rhijn and colleagues have shown that biological properties of AD-MSCs may not be affected by renal failure (22).

In this study we obtained MSCs from adipose tissue by means of lipo-aspiration from abdominal area. None of our patients encountered any mechanical catheter-related complication such as obstruction, hemoperitoneum, perforation, tunnel tract infection or leakage. Although, we were caution to obtain the samples from the opposite direction of the exit site. The two complications of peritonitis in case ID#3 in month 5 and exit site infection in case ID#4 in month 1 may not be attributed to the procedure, mostly because of the relatively long time interval between lipo-aspiration and the clinical manifestation. Moreover, our national registry data have shown that our average peritonitis rate was 1 episode in 25 patient-months (2). Our current results showed even a lower rate of peritonitis compared to our previous data, which might be due to close monitoring of the participants in this study. 

Similar to previous studies (23), we have shown the safety of AD-MSCs injection in human subjects, however our study had a particular importance, as we have assessed the safety of systemic infusion of MSCs in UFF patients. PD patients with UFF are a very specific group of highly sensitive patients and clinical treatment available for these patients is limited. It is obvious that protection of the peritoneal membrane or healing of a damaged membrane is of crucial importance for ESRD patients having no other alternatives except PD. Therefore, showing the feasibility and safety of MSC administration in these patients is essential for further studies assessing the efficacy of this treatment.

It is believed that the main mechanism in the induction of peritoneal fibrosis is mesothelial to mesenchymal transition that involves a complex process of cellular trans- differentiation (4). Moreover, angiogenesis and augmented vessel permeability also participate in increasing solute transport across the peritoneal membrane and UFF (24). In this study, we noticed a decline in the rate of solute transport across peritoneum as determined by standard PET and measured by D/Pcr.

This observation was similar to the results obtained in previous works performed in the same context (1317). Functionally, the number of perfused capillaries is suggested to be responsible for the fast dissipation of the osmotic gradient and high rate of transport across the membrane (24, 25). Brimble and colleagues, in a high quality meta-analysis demonstrated that a higher peritoneal membrane solute transport rate is associated with a higher mortality risk (26). Therefore, attenuation of the rate of solute transport across the membrane seen in this study following administration of MSCs is regarded as a positive change. Both *in vitro* and *in vivo* studies have reported that MSCs interact with a wide range of immune cells and suppress the excessive response of T cells, B cells, dendritic cells, macrophages, and natural killer cells, as well as induces regulatory T cells (Tregs) (10). MSCs have also been shown to maintain the capability of Tregs to suppress self-reactive T-effector responses (10, 27, 28). Although we cannot comment on the exact mechanism, by which MSCs exert this change, but the mentioned properties of stem cells for secreting the soluble factors crucial for cell survival and modulating the immune response might be responsible (29).

For future study design, we have to notice that our current study has some limitations. First, our study was not designed as a blind randomized controlled clinical trial, and therefore the changes seen after intervention cannot be exclusively associated with the intervention, as one might suggest that improvement of the rate of solute transport may be due to natural course of the disease. Second, since this was a clinical trial, the injected cells were not labeled, so we were not able to track their homing to the peritoneum. And third, because of the patients’ limitations, we did not follow up the patients for longer than six months. For a more sufficient outcome a longer follow-up period is desired for confirming the long term safety for chronic immunogenicity.

## Conclusion

This study showed for the first time that in PD patients systemic administration of AD-MSCs appears to be feasible and tolerated; at least over the six months follow- up period that we investigated. There might be some positive changes after this intervention in PD patients, however, there is certainly a need for further studies with larger sample sizes, more homogenous patients, longer follow-up periods, and control groups. Future investigations will need to elucidate the most effective route of administration, proper dose and frequency of MSC administration in PD patients. 
